# Adaptation to resistant hosts increases fitness on susceptible hosts in the plant parasitic nematode *Globodera pallida*

**DOI:** 10.1002/ece3.2079

**Published:** 2016-03-14

**Authors:** Sylvain Fournet, Delphine Eoche‐Bosy, Lionel Renault, Frédéric M. Hamelin, Josselin Montarry

**Affiliations:** ^1^ INRAUMR1349 IGEPP (Institute for Genetics, Environment and Plant Protection)F‐35653Le RheuFrance

**Keywords:** Experimental evolution, fitness benefit, life‐history traits, selection, trade‐off, unnecessary virulence

## Abstract

Trade‐offs between virulence (defined as the ability to infect a resistant host) and life‐history traits are of particular interest in plant pathogens for durable management of plant resistances. Adaptation to plant resistances (i.e.*,* virulence acquisition) is indeed expected to be associated with a fitness cost on susceptible hosts. Here, we investigated whether life‐history traits involved in the fitness of the potato cyst nematode *Globodera pallida* are affected in a virulent lineage compared to an avirulent one. Both lineages were obtained from the same natural population through experimental evolution on resistant and susceptible hosts, respectively. Unexpectedly, we found that virulent lineages were more fit than avirulent lineages on susceptible hosts: they produced bigger cysts, containing more larvae and hatching faster. We thus discuss possible reasons explaining why virulence did not spread into natural *G. pallida* populations.

## Introduction

The existence of trade‐offs between life‐history traits is a central concept in evolutionary biology and ecology because they may result in the wide range of species diversity that can be observed in nature. The trade‐off concept is based on a differential allocation of a limited resource to two different traits: maximizing one leads to reduced performance in the other (Stearns 1989). This concept provides an intuitive framework to explain why adaptation of populations to a new environment may be costly: selection of a highly beneficial trait in a new environment will be coupled to a cost in other trait(s).

Host–parasite interactions are particularly well suited to address experimentally the research questions relative to adaptation to a new environment, *that is* the host. Indeed, the increasing restrictions on pesticide use lead to the use of crop cultivars resistant to pathogens which represent an efficient, specific and environment friendly alternative strategy to control crop diseases. In this context, the overcoming of host specific resistance and the acquisition by the parasite of virulence, *that is* the ability to infect and multiply in host genotypes resistant to other parasite genotypes (Vanderplank 1963; Gandon and Michalakis 2002; Tellier and Brown 2007, 2009), represents an interesting case of host adaptation. Developing a sustainable use of plant resistances therefore requires exploring in detail the consequences of this adaptation on life‐history traits strongly involved in parasite fitness that can be in trade‐off with virulence.

Plant–parasite interactions often fit the widely accepted gene‐for‐gene model, which predicts that successful disease resistance is triggered only if a resistance gene product in the host recognizes, directly or indirectly, a specific avirulence gene product from the pathogen (Flor 1956, 1971; Jones and Dangl 2006; Brown and Tellier 2011). In such systems, a mutation from avirulence to virulence will increase pathogen fitness if the host resistant gene is present; many recorded examples of such directional selection, leading to the invasion of pathogen populations by virulent isolates, and consequently to resistance breakdown, have been reported (e.g., Vanderplank 1963; Wolfe 1993; Castagnone‐Sereno 2002; Molinari 2011; Brown 2015). Conversely, mutation to virulence can also involve strong fitness penalties in the absence of resistant hosts, and theoretical analyses of co‐evolution in gene‐for‐gene systems have shown that costs of virulence are necessary to maintain stable resistance‐avirulence polymorphisms (Brown and Tellier 2011).

Many efforts have been devoted to the experimental detection and quantification of virulence costs, and results are broadly in accordance with the predictions of a trade‐off model. Definitive evidence for resistance‐breaking costs has been reported, either in single infection or in competition, for several plant‐virus systems (Ayme et al. 2006; Sacristán and García‐Arenal 2008; Fraile and García‐Arenal 2010; Janzac et al. 2010; Poulicard et al. 2010, 2012; Fraile et al. 2011; Ishibashi et al. 2012; Montarry et al. 2012), bacteria (Leach et al. 2001) and fungi or oomycetes (Thrall and Burdon 2003; Abang et al. 2006; Huang et al. 2006; Bahri et al. 2009; Montarry et al. 2010). Plant parasitic nematodes are no exception to this rule. A reproductive fitness cost associated with virulence against the tomato *Mi‐1* resistance gene and against the pepper *Me3* and *Me7* resistance genes has been detected in the root‐knot nematode *Meloidogyne incognita* (Castagnone‐Sereno et al. 2007; Djian‐Caporalino et al. 2011), but there are no data regarding plant parasitic cyst nematodes. However, the overcoming of the resistance of potato cultivar Iledher, the first potato cultivar resistant to the cyst nematode *Globodera pallida* registered in the French catalogue, has been recently observed in an experimental evolution study (Fournet et al. 2013). The aim of the present work was to determine whether fitness costs of virulence are associated with this resistance breakdown, by studying life‐history traits on a susceptible host in a virulent/avirulent pair of lineages from a single *G. pallida* population. Both lineages were obtained through recurrent selection on a susceptible potato (cv. Désirée) for the avirulent one, and on a resistant potato (cv. Iledher) for the virulent one. To account for the adaptation dynamics, life‐history traits were studied in both lineages at the same time, from the sixth to the tenth generations.

## Methods

### Biology of *Globodera pallida*

*Globodera pallida* is a cyst nematode performing one generation for a vegetation season (Subbotin et al. 2010),*that is* only one generation per year in European climatic conditions. As other cyst nematodes, it enters plant roots as second‐stage juveniles (J2) and establishes a specialized feeding structure, the syncytium (Jones and Northcote 1972), which is a severe nutrient sink for the plant. Larvae mature and adult males then exit the roots in order to mate with females. After mating, the females continue to feed from the syncytium. When egg development is completed, and after an early moult of the first‐stage juveniles (J1) within eggs, females die and form a cyst, which protects the eggs until infective J2 hatch. The cyst, *that is* the body of the female, is the only structure which was transmitted from one season to the next, males acting only as gamete‐like propagules which do not survive.

### Selection of virulent and avirulent *Globodera pallida* lineages

Virulent and avirulent nematode lineages were established, as described by Fournet et al. (2013), starting from cysts of a French natural *G. pallida* population collected from an infested field (3 J2 per gram of soil) located near Saint‐Malo (Brittany, north‐western France), which has never been cultivated with any potato cultivar resistant to *G. pallida*. The lineages were obtained by rearing the nematode population during ten successive generations on either the resistant potato cultivar Iledher or the susceptible cultivar Désirée. Briefly, the first three generations were made directly into the field, the next two generations were made into 20 L tanks under greenhouse conditions and the five last generations into pots inoculated with 10 cysts coming from the previous cycle (see Fournet et al. 2013). The resistance of cv. Iledher depends on a major QTL, introgressed from *Solanum vernei*, explaining a large part (at least 61% – Rouppe van der Voort et al. 2000) of the genetic variance of segregating progenies and responsible for the development of most nematodes into adult males. This QTL was mapped on potato chromosome V and is designated as the *GpaVvrn* locus. The nematode lineages used here were Gp‐Désirée‐6x, Gp‐Désirée‐8x, Gp‐Désirée‐10x and Gp‐Iledher‐6x, Gp‐Iledher‐8x, Gp‐Iledher‐10x (x being the number of successive generations on the corresponding potato cultivar).

### Virulence assessment

**Figure 1:**
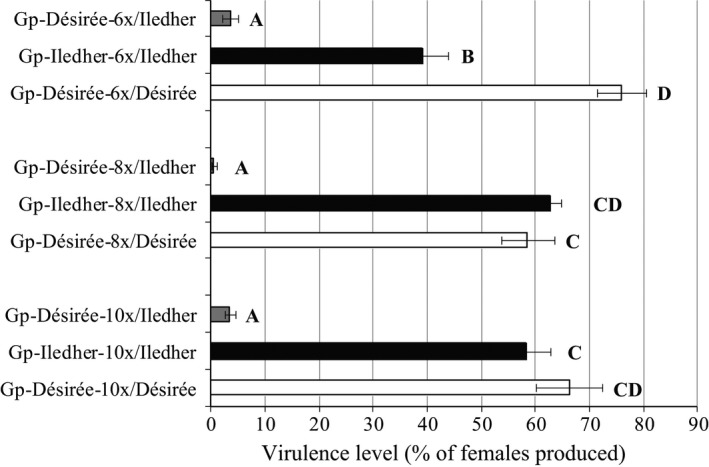
Percentage of females produced for each *Globodera pallida* lineage on the resistant potato cultivar Iledher and for the positive controls on the susceptible potato cultivar Désirée. Lineages having evolved on Désirée (Gp‐Désirée‐6x, Gp‐Désirée‐8x, Gp‐Désirée‐10x) and on Iledher (Gp‐Iledher‐6x, Gp‐ Iledher‐8x, Gp‐ Iledher‐10x) are indicated in grey and black, respectively. White bars correspond to the percentage of females produced by the three avirulent lineages (Gp‐Désirée‐6x, Gp‐Désirée‐8x and Gp‐Désirée‐10x) on Désirée. Letters represent the homogenous groups identified by the Tukey contrasts test at the 5% threshold.

Response to selection was measured by estimating the virulence level, defined as the probability for a larva to produce a female, in each lineage. The virulence of a given lineage was estimated as described by Fournet et al. (2013). Briefly, pieces of germinated tubers were allowed to emit roots in a petri dish containing 20 g·L^−1^ water agar, and one root was inoculated with ten newly hatched larvae coming from a pool of ten cysts placed into potato root exudates. After inoculation, petri dishes were kept in total darkness, under controlled conditions (20 ± 1°C), for 15 days. Every inoculated root was then dissected in water under a transillumination unit for brightfield/darkfield contrasts, and the number of developed females was counted. Virulence level was then calculated for each lineage as the ratio between the number of females and inoculated larvae. Virulence level of each nematode lineage was assessed in this way on the resistant potato cultivar Iledher and compared for each generation to the corresponding percentage of females produced by the avirulent lineages (Gp‐Désirée‐6x, Gp‐Désirée‐8x, Gp‐Désirée‐10x) on the susceptible potato cultivar Désirée. This comparison allowed us to define the virulence status of each lineage (“vir” for virulent or “avir” for avirulent; see Fig. 1). For each lineage, from 12 to 29 roots (*i.e.,* replicates) were inoculated independently.

### Life‐history traits assessment

Life‐history traits were evaluated on new cysts produced on the susceptible potato cultivar Désirée, under controlled conditions, using larvae issued from the lineages Gp‐Désirée‐6x, Gp‐Iledher‐6x, Gp‐Désirée‐10x and Gp‐Iledher‐10x. To this end, germinated tubers of the cultivar Désirée were buried in a moistened mixture of sand and soil (1:3) contained in a 150‐mL clear plastic screw cup (C.E.B., Angers, France). Capped cups were then stored in the dark at 20°C (± 1) and 70% relative humidity in a climatic chamber until roots were clearly visible through the cup wall. Thereafter, for each lineage, six cups (*i.e.,* six independent replicates) were inoculated with 2 mL of a suspension containing around 150 second‐stage juveniles·mL^−1^. This inoculum suspension was obtained by pooling together the larvae collected every 4 days during a maximum of 12 days from 80 different cysts soaked in potato exudates (cv. Désirée). Freshly hatched larvae were stored at 4°C until the proper inoculum concentration was reached. The final concentration was adjusted by adding the corresponding amount of water and controlled five times by counting all the larvae in 1 mL just before inoculation. Cups were then capped again and stored for 80 days in the climatic chamber, under the same climatic conditions, to allow the complete development and maturation of newly formed cysts.

For each replicate, newly formed cysts were manually collected and numbered (#Cyst) after extraction with a Kort elutriator. The size of each collected cyst (Size_cysts) was evaluated through the surface area using a magnifying stereomicroscope coupled with an image analysis software (Microvision Instruments, Histolab v8.1.0, Evry, France). To evaluate the cysts' contents, ten cysts were randomly picked from each replicate, pooled and crushed in water, and the eggs and larvae were counted (#Larvae) using a magnifying stereomicroscope. For each replicate, *that is* for each pool of ten cysts, the arc length of ten randomly chosen larvae was measured (Length_larvae) using a magnifying stereomicroscope coupled with an image analysis software (Microvision Instruments, Histolab v8.1.0, Evry, France). To estimate the hatching dynamics, ten other cysts, randomly picked from each replicate, were soaked in potato exudates (cultivar Désirée) in a small plastic cup and then stored in the dark at 20°C (± 1). Every 5 days, the potato exudate was removed and the hatched larvae were counted using a magnifying stereomicroscope. Seventy days after the start of the experiment, cysts were crushed in water to assess the number of unhatched larvae, so as to calculate the final hatching rate (a proportion) of each replicate. Assuming that the time‐to‐hatching is Weibull‐distributed, a simple model (Data S1) was adjusted to the hatching curves and two parameters, the hatching probability (P) and the mean hatching time (HT), were estimated for each replicate, through a maximum likelihood approach.

### Statistical analysis

All statistical analyses were performed using the R software version 3.1.1 (R Core Team, 2014). Normality and homogeneity of variances were checked using the Shapiro–Wilk and the Leven tests, respectively.

The levels of virulence of each lineage (Gp‐Désirée‐6x, Gp‐Désirée‐8x, Gp‐Désirée‐10x and Gp‐Iledher‐6x, Gp‐Iledher‐8x, Gp‐Iledher‐10x) and of the positive controls (corresponding to the percentage of females produced by the three avirulent lineages, Gp‐Désirée‐6x, Gp‐Désirée‐8x, Gp‐Désirée‐10x, on the susceptible potato cultivar Désirée) were compared with a one‐way ANOVA on the percentage of females produced and multiple comparisons of means with the Tukey contrasts test (*α *= 0.05).

The pairwise relationships between the different life‐history traits measured (#Cyst, Size_cysts, #Larvae, Length_larvae, P and HT) were separately tested on the virulent and avirulent lineages with the Pearson's product–moment correlation. Significance of the correlations was corrected for multiple comparisons using the p.adjust R function and the false discovery rate (FDR) method developed by Benjamini and Hochberg (1995).

For each life‐history trait (#Cyst, Size_cysts, #Larvae, Length_larvae, P and HT), the generation effect, the vir/avir effect (*i.e.,* the virulent/avirulent status of lineages) and the corresponding two‐way interaction effect were tested through an ANOVA. Moreover, for each generation (6x and 10x), the vir/avir effect was tested for the six life‐history traits using a one‐way ANOVA.

## Results

### Virulence level of the different *Globodera pallida* lineages

The ANOVA model showed a significant lineage effect on the percentage of females produced (F_8,161_ = 69.56; *P *<* *0.0001). *Globodera pallida* lineages having evolved on the susceptible potato cultivar Désirée, during either six, eight or ten generations, showed a very low virulence level on the resistant potato cultivar Iledher (from 0.5% to 3.5% of females produced – Fig. 1). That result highlighted the high efficacy of that resistance, conferred to Iledher by the *GpaVvrn* QTL, and confirmed the avirulent status of those lineages, named hereafter avir(6x), avir(8x) and avir(10x). Conversely, *G. pallida* lineages having evolved on cultivar Iledher showed a high virulence level on that cultivar, confirming the virulent status of those lineages, named hereafter vir(6x), vir(8x) and vir(10x). While after six generations, the virulence level reached 38.9% of females produced, indicating that the breakdown of resistance was incomplete (*i.e.,* significantly different from the corresponding control – Fig. 1), it reached 62.5% and 58.1% of females produced after eight and ten generations, respectively, indicating that the breakdown was total (*i.e.,* not significantly different from their controls – Fig. 1). Because virulence levels from avir(8x) and vir(8x) on the one hand and avir(10x) and vir(10x) on the other hand were not significantly different; the comparisons of life‐history traits between virulent and avirulent lineages were made only using 6x and 10x *G. pallida* lineages in order to perform the fitness cost test.

### Life‐history traits measurements in virulent and avirulent lineages

There was no significant correlation between the different life‐history traits measured here, *that is* number of cysts, size of cysts, number of larvae per cyst, length of larvae, hatching probability and mean hatching time, whatever the status of the *G. pallida* lineages (Table 1A for the virulent and Table 1B for the avirulent lineages, respectively). Note that there was also no significant correlation between the different life‐history traits when the test was done separately for each lineage (data not shown). Consequently, there was no trade‐off between the different life‐history traits measured here, and because there was no redundant information, comparisons between virulent and avirulent lineages were relevant for all six life‐history traits.

**Table 1:** Pearson's correlation coefficients (below the diagonal) and associated adjusted *P*‐values (above the diagonal) between the different life‐history traits (#Cyst, Size_cysts, #Larvae, Length_larvae, P and HT) for (A) the virulent and (B) the avirulent *Globodera pallida* lineages

	#Cysts	Size_cysts	#Larvae	Length larvae	P	HT
(A)						
#Cysts		0.671	0.780	0.671	0.397	0.813
Size_cysts	−0.27		0.671	0.397	0.671	0.671
#Larvae	−0.11	−0.35		0.780	0.397	0.780
Length_larvae	0.26	−0.60	0.12		0.671	0.397
P	−0.49	0.34	0.54	−0.23		0.671
HT	−0.08	−0.29	0.13	0.49	−0.22	
(B)						
#Cysts		0.779	0.514	0.606	0.779	0.281
Size_cysts	−0.19		0.779	0.375	0.281	0.841
#Larvae	0.36	−0.14		0.293	0.386	0.873
Length_larvae	−0.30	0.46	0.55		0.281	0.375
P	0.16	0.63	0.44	0.59		0.779
HT	0.62	−0.09	0.05	−0.47	0.20	

**Figure 2:**
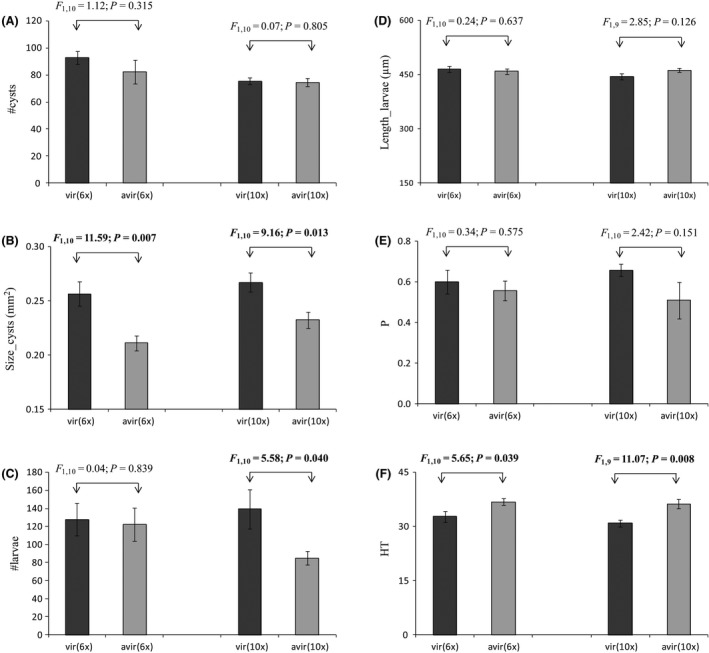
Means (± standard error) of (A) the number of cysts (#Cysts), (B) the size of cysts (Size_cysts), (C) the number of larvae (#Larvae), (D) the length of larvae (Length_larvae), (E) the hatching probability (P) and (F) the mean hatching time (HT) for the virulent and the avirulent *Globodera pallida* lineages at each generation (6x and 10x). *F* and *P* are indicated for each comparison and significant vir/avir effects are highlighted in bold.

The two‐way ANOVAs revealed (1) a small generation effect for the number of cysts (with more cysts produced at the sixth generation than at the tenth generation), (2) a vir/avir effect for the size of cysts and the mean hatching time and (3) no significant interaction effect (Table 2). The number of cysts produced on the susceptible cultivar potato Désirée did not differ significantly between the virulent and the avirulent *G. pallida* lineages (Fig. 2A). However, cysts were significantly larger for the virulent than for the avirulent lineages, regardless of the generation considered (Fig. 2B). The virulent lineage produced more larvae per cyst than the avirulent lineage at the tenth generation, and there was no effect on that life‐history trait at the sixth generation (Fig. 2C). Those larvae were of similar length in the virulent and the avirulent lineages (Fig. 2D). Finally, the mean hatching time was significantly longer in the avirulent than in the virulent lineages (Fig. 2F and Data S2), whereas there was no significant difference for the hatching probability, whatever the generation considered (Fig. 2E and Data S2).

**Table 2:** Results from the analyses of variance (ANOVas) assessing the generation effect (6x and 10x), the vir/avir effect and the corresponding two‐way interaction of these factors on the six life‐history traits (#Cyst, Size_cysts, #Larvae, Length_larvae, P and HT). The statistically significant effects are indicated as *P <* *0.05, ***P <* *0.001

Source of variation	#Cysts			Size_cysts			#Larvae			Length_larvae			P			HT		
	df	*F* value	*P* > *F*	df	*F* value	*P* > *F*	df	*F* value	*P* > *F*	df	*F* value	*P* > *F*	df	*F* value	*P* > *F*	df	*F* value	*P* > *F*
Generation effect	1	5.34	0.032*	1	3.32	0.084	1	0.56	0.461	1	1.22	0.284	1	0.01	0.939	1	1.09	
Vir/avir effect	1	1.16	0.294	1	20.74	0.0002***	1	2.98	0.100	1	0.45	0.509	1	2.51	0.129	1	16.10	0.0007***
Interaction effect	1	0.80	0.382	1	0.35	0.560	1	2.00	0.173	1	2.15	0.159	1	0.76	0.393	1	0.31	0.584
Error	20			20			20			19			20			20		

## Discussion

Evaluating life‐history trade‐offs associated with unnecessary virulence in plant pathogens is the classical method to demonstrate that selection for virulence could be costly. This point is central to understand the ecology and evolution of plant diseases, and thus to better manage plant resistances on the long run. According to the trade‐off concept, resistance‐breaking isolates are expected to undergo a fitness cost in susceptible hosts, compared to avirulent isolates. This is true for many plant pathogens such as viruses, oomycetes and fungi (see Laine and Barrès 2013 for a review), but also in the case of root‐knot nematodes (Castagnone‐Sereno et al. 2007; Djian‐Caporalino et al. 2011). Unexpectedly, in our experimental conditions, we found an apparent fitness benefit for virulent lineages in *G. pallida*, even on the susceptible host: the virulent lineages produced bigger cysts, containing more larvae (at least for the tenth generation) and hatching more quickly. Generalization of that result could be reinforced using other pairs of virulent and avirulent lineages coming from other natural cyst nematode populations.

The lack of apparent cost related to virulence is a result which might bear several explanations. First, the resistance conferred by *GpaVvrn* basically alters the quality of the feeding site, and thus acts on a late step of the nematode development cycle (Fournet et al. 2013). Therefore, the traits involved in the early stages of parasitism could be less affected by selection against unnecessary virulence. This seems to be true as the same number of cysts for both lineages was observed, suggesting that virulent larvae exhibit the same abilities as avirulent ones to localize and then penetrate inside the roots, to move within the roots, to build their feeding site and to develop into adult males and females. Accordingly, Castagnone‐Sereno et al. (2015) recorded no cost related to unnecessary virulence in life‐history traits directly linked to the establishment of the parasitic interaction between tomato and *M. incognita*. Another possible explanation is that virulence costs were compensated before our first measurement of a potential cost, *that is* before the sixth generation. For example, Schoustra et al. (2006) showed that fludioxonil‐resistant isolates of the ascomycete *Aspergillus nidulans* suffered a severe fitness cost in the absence of the drug, but that this cost was afterwards waived without loss of the resistance. In the contrary, Janzac et al. (2010) performed a serial passage experiment with potato virus Y in pepper and observed no compensatory mutation showing that the only possibility to compensate a high virulence cost was through the reversion from virulence to avirulence. Because no *G. pallida* cyst was sampled before the sixth generation, we cannot actually test the hypothesis of an early cost compensation.

Finding that a reproductive fitness benefit (*i.e.,* an increase in the ability of virulent individuals to produce a progeny) is associated with unnecessary virulence is much more surprising than the apparent absence of a cost only. To our knowledge, such an unexpected result has been previously recorded only twice, in the wheat pathogen *Puccinia striiformis* f. sp. *tritici*, for which the *vir9* virulence factor conferred a competitive advantage in the absence of the corresponding resistance gene (Bahri et al. 2009), and for one life‐history trait of the potato late blight pathogen, *Phytopththora infestans*, where virulent isolates had a shorter latency period than avirulent ones (Montarry et al. 2010). However, for the former, this was not true for two other virulence factors (*vir4* and *vir6*), and for the latter this advantage was counterbalanced by costs for other life‐history traits leading to an overall fitness lower for virulent isolates compared to avirulent ones on susceptible hosts.

The positive relationship between female body size and fecundity is a well‐known correlation, particularly in ectotherms: larger females will produce more eggs (e.g., Reiss 1989; Roff 1992), and this has already been highlighted in nematodes (Morand 1996). In our study, virulent females had a larger body size at the sixth and tenth generations (compared to the avirulent lineage). This may be the consequence of an adaptation to the difficulty to build high‐quality feeding sites in the resistant cultivar during the experimental evolution. This may correspond to the “thrifty phenotype” hypothesis developed by Hales and Baker (2001) which asserts that nutrient stress during early development leads to an adapted physiological state that increases fitness in conditions of food scarcity. Such theory was recently confirmed in the free‐living nematode *Caenorhabditis elegans* where the progeny and grand progeny of starved larvae are more resistant to starvation (Jobson et al. 2015). Accordingly, one can imagine that this adaptation may lead to a more efficient use of the amount of available resources in susceptible host plants by virulent females, leading to larger body sizes and offspring number, which represents an obvious fitness gain.

The offspring of virulent females moreover hatches faster than the offspring of avirulent females. Here, it is less easy to determine whether it can give a fitness advantage or not as *G. pallida* achieves only one generation per year. Nevertheless, one can imagine that hatching more quickly allows the larvae to penetrate in better quality roots (*i.e.,* not parasitized by a large number of competitive larvae), and should enhance their development success. Moreover, in the case of mixed populations where virulent and avirulent larvae compete, such hatching differences could promote mating between virulent females and virulent males.

If the present work attested that the adaptation to the *GpaVvrn* QTL involves no cost of virulence but rather provides a fitness advantage, it remains unclear why that virulence mutation did not spread into natural *G. pallida* populations. This question is particularly relevant as the most parsimonious hypothesis is that the mutation has to be already present in the population to allow adaptation to resistance. Indeed, the fact that some nematode lineages were lost during the experimental evolution study performed by Fournet et al. (2013) constitutes an argument that few individuals carrying the virulence alleles are present in natural populations. Even if the virulence seems to be present in some populations and advantageous, the system is probably more complex in natural conditions than in our laboratory experiments. Indeed, gene flow, which was zero during our experimental evolution experiment but often strong in agro‐ecosystems due to passive transport of cysts through human activities (Picard et al. 2004; Plantard and Porte 2004; Alenda et al. 2014), and small effective population size (Jan et al. 2016), which favours the random action of genetic drift, can both strongly reduce the frequency of virulence alleles in natural populations. Another possible explanation could be that trade‐offs between virulence and fitness traits exist in the field situation but were overshadowed in our experimental situation, Moreover, the competition between virulent and avirulent individuals which takes place in natural populations could lead to the emergence of virulence costs on traits that have not been studied here (e.g., survival abilities when the host is absent). Several experiments on fungi and viruses have studied such a competitiveness cost by performing competition experiments involving infections with mixture of virulent and avirulent isolates (Abang et al. 2006; Bahri et al. 2009; Janzac et al. 2010) and highlighted in most cases a competitiveness cost leading to the counter‐selection of virulent individuals on the susceptible host.

To complement our results, further competition experiments should be performed by studying the evolution of frequencies of *G. pallida* virulent and avirulent variants on a susceptible potato cultivar. However, this kind of experiment requires genetic markers to follow the virulent/avirulent alleles. Such markers are not yet available for *G. pallida*, but studies are currently performed to develop them. Elucidating the genetic determinism of virulence will also allow insight into whether the gene(s) involved is(are) present in multicopy, which could explain the absence of cost, as a mutated copy (virulent) could be associated with a nonmutated copy (avirulent) which could assure the initial function of the gene. Obviously, the use of different *G. pallida* populations, selected on different genotypes carrying the *GpaVvrn* QTL, should allow our conclusions to be extended and is of a crucial importance to address sustainable management of resistance.

## Data Archiving

All data used in this article are available at datadryad.org (http://dx.doi.org/10.5061/dryad.j9j02).

## Conflict of Interest

None declared.

## Supporting information


**Data S1.** Nematode females produce cysts which contain eggs, which may hatch and produce larvae.
**Data S2.** Percentage of hatching (± standard error) measured during 70 days with a five‐day interval (A) for the virulent and avirulent lineages after six generations and (B) for the virulent and avirulent lineages after ten generations.
