# Supplementary material for: Adaptation to resistant hosts increases fitness on susceptible hosts in the plant parasitic nematode Globodera pallida
Source: Ecol Evol. 2016 Mar 14;6(8):2559–68. doi: 10.1002/ece3.2079 (PMC4797161; doi:10.1002/ece3.2079)
Supplement: Supplementary file 1 — Data S1. Nematode females produce cysts which contain eggs, which may hatch and produce larvae. Data S2. Percentage of hatching (± standard error) measured during 70 days with a five‐day interval (A) for the virulent and avirulent lineages after six generations and (B) for the virulent and avirulent lineages after ten generations. [file ECE3-6-2559-s001.docx]

**Supporting information S1**

Nematode females produce cysts which contain eggs, which may hatch and produce larvae.

We aim to model the dynamics of larvae production to better compare virulent and avirulent lineages obtained from experimental evolution on resistant and susceptible host genotypes, respectively.

**Data** We have

- the times at which newly hatched larvae are collected and counted:

$\left\{ t_{i} \right\}=\left\{ t_{0},t_{1},t_{2},\cdots,t_{k} \right\}$,

- the number of larvae corresponding to each observation time:

$$\left\{ n_{i} \right\}=\left\{ n_{0},n_{1},n_{2},\cdots,n_{k} \right\}.$$

- At the end of the experiment (time $t_{k}$), cysts are dessicated and unhatched eggs are counted: let their number be $n_{u}$, so $n_{u}+\sum_{i=0}^{k} n\_i=N$is the total number of eggs or potential larvae.

We are interested in the proportion of hatched larvae as a function of time, say $H(t)$. Let $H_{d}\left( t_{i} \right)=\sum_{j=0}^{i} n_{j}/N$ be the observed dynamics.

**Model** We assume that each egg is viable with probability $P$ (hatching probability). We also assume that the time-to-hatching is a random variable $T$ with a cumulative distribution $F(t)$ which depends on the parameter $\Theta$, and which support is $\left[ 0,+\infty) \right.$. Let $\theta=\{P,\Theta\}$ be the set of parameters to be estimated. Also, let $H_{\theta}\left( t \right)=PF(t)$ be the theoretical proportion of hatched larvae.

The probability to observe the data given a parameter set $\theta$ (its likelihood) is

$$\begin{matrix} L\left( \theta\right) & = & \underset{\text{Probability}(n_{0}\text{ hatched before }t_{0})}{\underbrace{\left( \begin{matrix} N \\ n_{0} \end{matrix} \right){H_{\theta}\left( t_{0} \right)}^{n_{0}}\left( 1-H_{\theta}\left( t_{0} \right) \right)^{N-n_{0}}}} \\ & \times& \prod_{i=1}^{k} \underset{\text{Probability(}n_{i} \text{hatched between }t_{i-1}\text{ and }t_{i} | \text{they did not hatch before)}}{\underbrace{{\binom{N-\sum_{j=0}^{i-1} n_{j}}{n_{i}}\left( \frac{H_{\theta}(t_{i})-H_{\theta}(t_{i-1})}{1-H_{\theta}(t_{i-1})} \right)}^{n_{i}}\left( 1-\frac{H_{\theta}(t_{i})-H_{\theta}(t_{i-1})}{1-H_{\theta}(t_{i-1})} \right)^{N-\sum_{j=0}^{i} n_{j}}}} \end{matrix}$$

Because the combinations do not depend on $\theta$, we define a log-likelihood function as

$\begin{matrix} LL\left( \theta\right) & = & n_{0}\log\left( H_{\theta}\left( t_{0} \right) \right)+{(N-n}_{0})\log\left( 1-H_{\theta}\left( t_{0} \right) \right) \\ & + & \sum_{i=1}^{k} \left[ n_{i}\log\left( \frac{H_{\theta}(t_{i})-H_{\theta}(t_{i-1})}{1-H_{\theta}(t_{i-1})} \right)+\left( N-\sum_{j=0}^{i} n_{j} \right)\log\left( 1-\frac{H_{\theta}(t_{i})-H_{\theta}(t_{i-1})}{1-H_{\theta}(t_{i-1})} \right) \right] \end{matrix}$ .

Assuming that the time-to-hatching is Weibull-distributed, we let $\Theta=\{\lambda,k\}$ and

$F\left( t \right)=1-e^{-\left( \frac{t}{\lambda} \right)^{k}}$.

The mean hatching time $HT$ can then be expressed through a relationship involving the Gamma function:

$$HT=\lambda\Gamma\left( 1+\frac{1}{k} \right).$$

**Supporting information S2** Percentage of hatching (± standard error) measured during 70 days with a five-day interval [**A**] for the virulent and avirulent lineages after six generations and [**B**] for the virulent and avirulent lineages after ten generations. Lines represent the model adjusted to the hatching data for each lineage.
